# Obituary

**Published:** 1912-01-13

**Authors:** 


### Obituary.

Dr. James Peddie Harper, M.D., has just died at the age of eighty-five. He was one of the oldest medical men alive, having qualified in 1848 at the University of Edinburgh. Dr. Harper held appointments at the Royal Infirmary, Dumfries, and at Leith Fever and Casualty Hospital, where he was one of the physicians. He was formerly connected with Windsor, and his funeral took place at the Windsor cemetery.

Mr. John Swindale, M.R.C.S., has died at Ipswich at the age of sixty-nine. He was formerly in active practice at Binfield, near Reading, Berkshire, where he remained for about thirty-five years. He qualified in 1864 from the Middlesex Hospital, at which he held the posts of Resident Physician and Registrar. Mr. Swindale then became House Surgeon at the Royal Berkshire Hospital, Reading, and took an active part in the well-known pathological Society of that town. During the last ten years he lived in retirement at Ipswich, but continued to devote a large portion of his leisure to various philanthropic societies and to the Ipswich Clinical Society. He leaves two sons in the medical profession, and one of his daughters has an established position in the nursing world.

